# A Retrospective Analysis of Self-Limiting Fever following Percutaneous Patent Foramen Ovale and Atrial Septal Defect Closure

**DOI:** 10.1155/2024/5562208

**Published:** 2024-10-09

**Authors:** Francesca Galasso, Felicia Wassenaar, Timothy Barry, Omar J. Baqal, Donald J. Hagler, John P. Sweeney, F. David Fortuin

**Affiliations:** ^1^Department of Cardiovascular Diseases, Mayo Clinic Arizona, 5777 E Mayo Boulevard, Phoenix 85054, AZ, USA; ^2^Department of Internal Medicine, Mayo Clinic Arizona, 5777 E Mayo Boulevard, Phoenix 85054, AZ, USA

## Abstract

While percutaneous closure of patent foramen ovale (PFO) and atrial septal defect (ASD) are generally well-tolerated procedures, the development of postprocedure fever has been observed at a higher frequency than reported in the initial device trials. We performed a retrospective analysis of 62 patients who underwent PFO or ASD closure from January 1, 2020, to December 31, 2022, at Mayo Clinic, Arizona. Eight patients out of 62 (12.9%) developed fever following PFO or ASD closure. In each of the fever cases, the Gore Cardioform devices (W.L. Gore and Associates, Flagstaff, AZ) were used. No association was found between clinical characteristics or procedural details and the development of fever. The reactions occurred 24 to 48 hours following device implantation and resolved spontaneously. No evidence of infection was found upon diagnostic evaluation. There was a higher incidence of self-limited atrial fibrillation (AF) in the fever patients (37.5% vs. 18.5%) which was not statistically significant. All patients who developed fever had successful closure with no other subsequent clinical events. We have found a high incidence of fever following PFO or ASD closure using the Gore family of devices that has not been observed in prior years. A unifying etiology or risk factor, such as infection or medication, for the fever could not be identified. Long-term device success was achieved in all fever patients. This small retrospective study suggests that the observed fever is benign and self-limiting but further investigation is warranted to determine its true incidence, mechanism, and prognosis.

## 1. Introduction

Transcatheter closure of atrial septal defect (ASD) and patent foramen ovale (PFO) are commonly performed procedures with high success rates and a low incidence of complications [1–3]. Serious adverse events associated with percutaneous closure include device embolization, erosion, and new-onset atrial arrhythmias. However, fever has been rarely reported as an adverse reaction. The clinical studies that assessed the safety and efficacy of the Gore Cardioform Septal Occluder (GSO) device in the REDUCE trial and the Gore Cardioform ASD Occluder (GCA) device (W.L. Gore and Associates, Flagstaff, AZ) in the ASSURED trial, listed fever as occurring in less than 1% of cases [1, 3]. However, at our institution, Mayo Clinic Arizona, we have recently observed a seemingly higher incidence of fever following the use of these devices. To better understand this perceived, we conducted a systematic retrospective review of all PFO and ASD closure cases performed at our institution from January 1, 2020, to December 31, 2022.

## 2. Materials and Methods

This was a single-center, retrospective chart review study conducted on patients who underwent PFO or ASD closure at Mayo Clinic, Arizona, from January 1, 2020, to December 31, 2022. The study was approved by the Mayo Clinic Institutional Review Board. The inclusion criteria for the study were all adult patients (age ≥18 years) who underwent successful transcatheter PFO or ASD closure at our institution from January 2020 to December 2022. Patients who developed transient fever within the first week postprocedure were compared to patients who did not develop fever. Fever (including low-grade fever: 99.1 to 100.4 degrees Fahrenheit) [4] was either self-reported or documented in the medical record.

For descriptive statistics, continuous variables were expressed as mean and standard deviation (SD). For the comparison of two continuous variables with normal distribution, a *t*-test was used. For the comparison of three or more continuous variables with normal distribution, an ANOVA test was used. For categorical variables, Fisher's exact or chi-square test was used. All tests were two-sided, and a significance level of *p* < 0.05 was established. Statistical analysis was performed in Microsoft Excel and BlueSky Statistics software v. 7.40 (BlueSky Statistics LLC, Chicago, IL, USA).

## 3. Results

### 3.1. Patient Characteristics

A total of sixty-two patients (mean age 57.8 years, 50% male) who had successful PFO or ASD closure between January 1, 2020, and December 31, 2022, were identified and included in the study. Eight patients (12.9%) had fever postprocedure. There were no statistically significant differences in patient demographics, previous medical and social history, and current medications between the two groups. One patient in the nonfever group has a nickel allergy and no patients in the fever group have a nickel allergy (Table 1).

### 3.2. Details on the Patients Who Developed Fever

A total of eight patients out of 62 (12.9%) developed fever following PFO or ASD closure. The average time to develop fever following the procedure was 1.25 days. The range of maximum temperature (*T*_max_) was 99.9 F–101.7 F with an average *T*_max_ of 100.7 F. Every patient in the fever group was implanted with a Gore Cardioform device: 6 received the Gore Cardioform septal occluder (GSO) and 2 received the Gore Cardioform ASD occluder (GCA). Six out of the 8 patients underwent laboratory and radiographic evaluation to determine a possible source of infection. All tests including blood culture, urinalysis, chest X-ray, COVID-19, and influenza testing were negative. Detailed results are shown in Table 2. Patient 1 self-reported fever and did not disclose temperature. Patient 4 reported procalcitonin count of <0.06. Patient 5 had an erythrocyte sedimentation rate (ESR) of 26 and C-reactive protein of 24.6 mg/L with normal chest X-ray and ECG results. She received 3 grams of cefazolin in the hospital postprocedure after developing fever overnight. Patient 7 had a C-reactive protein of 64.0 mg/L with normal chest X-ray and ECG results. Six out of the eight patients in the fever group (75%) were fully vaccinated against COVID-19 at the time of the reaction with three out of the eight (37.5%) having received a 3rd dose. Patients in the fever group also reported symptoms such as chills, headache, body aches, nausea, and chest tightness. After three months postprocedure, five out of eight patients received a transesophageal echocardiogram (TEE), two received a transthoracic echocardiogram (TTE), and one had not received a follow-up echocardiogram.

### 3.3. Preprocedure Data

Preprocedure echocardiographic and transcranial Doppler (TCD) ultrasonographic data were collected to characterize the defect of PFO or ASD and evaluate shunt size (Table 3). The preprocedure data between the two groups were similar, and no significant differences were observed.

### 3.4. Procedural Details

Within the fever group, 62.5% were PFO closure and 37.5% were ASD closure. The indications for the procedure in the fever group were secondary prevention of stroke/TIA (4/8, 50%), ASD (3/8, 37.5%), and hypoxia (1/8, 12.5%). 6 patients (75%) received the GSO device and 2 (25%) received the GCA device. None of the patients who reported fever received an Amplatzer device. Only 3 Amplatzer devices were used over the study period. Six patients were discharged on the same day of the procedure while 2 were observed overnight and discharged the next day. Three patients in the fever group underwent their procedure in 2022 (37.5%), 4 in 2021 (50.0%), and 1 in 2020 (12.5%) The mean follow-up duration was 13.3 ± 9.4 months (range, 1–37 months) with 14.3 months of follow-up in the fever group and 13.2 months in the nonfever group (Table 4). For procedure sedation and guidance, two patients in the fever group (25.0%) and 7 in the nonfever group (13.0%) were under general anesthesia. For the remaining, moderate sedation was used. Regarding guidance, intracardiac echocardiography (ICE) was used for all cases with moderate sedation. Transesophageal echocardiography (TEE) or TEE and ICE combined was used for cases with general anesthesia. For the two patients under general anesthesia in the fever group (25.0%), one used TEE guidance and the other TEE + ICE guidance. Of the seven patients under general anesthesia in the nonfever group (13.0%), 6 used TEE guidance and one TEE + ICD guidance. There are no statistically significant differences between the fever group and nonfever group in indication, device type, device size, max ACT, date of discharge, postprocedure ED admission, type of sedation, type of guidance, year of procedure, and closure rate.

### 3.5. Adverse Events

Atrial fibrillation (AF) was numerically more frequently observed in the fever group compared to the nonfever group (3/8, 37.5% vs. 10/54, 18.5%; *p* value = 0.347; Table 5). The total occurrence of AF in this study is 20.9% (13/62). The mean time to develop postprocedure AF was 12.0 ± 5.3 days for the fever group and 16.2 ± 8.1 days for the nonfever group (*p* value = 0.425). Adverse events including AF, urinary symptoms, gastrointestinal symptoms, respiratory symptoms, and death did not differ significantly between the two groups. No deaths occurred in the fever group and two deaths occurred in the nonfever group (one fatal myocardial infarction 228 days postimplantation and one likely fatal infection 109 days postimplantation at an outside institution with no additional details available).

## 4. Discussion

This study evaluated a cohort of patients who had undergone recent PFO or ASD closure at Mayo Clinic, Arizona, evaluating the incidence of postprocedure fever and determining if there is a correlation among patient characteristics, procedural details, and outcomes. Despite the reported rare (<1%) incidence of fever reported in randomized trials [1, 3], we observed a relatively frequent occurrence (12.9%). Baseline characteristics and procedural details did not offer any explanation for the fever. We also reviewed the clinical details of each fever patient such as device used, maximum temperature, blood workup results, and more. All patients who developed fever tested negative for infection and had a complete recovery. In the patients who developed fever, there was a 100% procedural success and a 100% long-term device success rate at follow-up.

No infectious etiology was found in any of the patients who experienced postprocedure fever. With no evidence of sepsis or infection, these patients may be experiencing an inflammatory response to the device. Previous reports involving the BioStar (NMT Medical Inc., defunct) device discuss a similar hypothesis for the etiology of these fevers. In 2009, a report from Milan, Italy, described two patients who developed transient fevers 24 hours after the implantation of the BioStar device. There was no clinical evidence of infection, and the fevers resolved quickly with steroidal treatment. The researchers hypothesized that these patients are exhibiting a systemic reaction to the acellular porcine collagen matrix within the BioStar device [5]. Similarly, a 2020 study carried out in Slovenia came to a similar conclusion. They found an incidence of 10.8% of patients with the BioStar device developing transient fever and speculated that these patients are reacting to the device's collagen membrane [6]. The patients in these studies had a similar clinical presentation to those involved in our study, but instead with the use of Gore Cardioform devices. Interestingly, the Gore occluders consist of a membrane made of expanded polytetrafluoroethylene (ePTFE), a polymer material, and thus, lack the porcine collagen membrane that was hypothesized to cause these fevers. In addition, our communication with W.L. Gore and Associates, Inc., confirmed that every device implanted in the patients who developed fever or chills passed sterilization records with no alterations to the process (personal communication). It is ultimately unclear if the occluder itself is responsible for these reactions.

While the etiology of these postprocedure fevers is not well understood, their presence may serve as a predictor of an adverse cardiac event, such as AF. There was a numerically higher incidence of AF in the patients who had also developed a fever compared to those who did not, albeit the difference was not statistically significant. AF is a known complication that follows PFO or ASD closure that is usually paroxysmal and self-limiting. It is possible that the procedure and/or device can cause atrial irritation that can induce an inflammatory response in the cardiac tissue which could trigger atrial arrhythmia [7, 8]. This is consistent with our postulation that device implantation produces a systemic inflammatory response presenting as a transient fever. The presence of a fever may be indicative of an inflammatory reaction occurring in the body and thus can predict the development of AF. This may help patients and their providers in taking proactive measures to monitor for AF.

We observed a higher incidence of AF than that reported in trials (6.6% in REDUCE Trial [1] vs. 13 of 62 (20.9%) in this study). This may be due to the younger patient population in prior trials compared to this study (45.2 years vs. 57.8 years). The reported incidence of AF postprocedure in the literature is highly variable and may be related, at least in part, to the extent of AF monitoring postprocedure [7, 9].

Limitations of the study include the retrospective design and relatively small sample size.

Despite not finding a cause for fever in our patients, we still find this incidence of postprocedure fever or chills compelling. Fever, chills, or any other inflammatory response following a procedure is alarming as it could be indicative of a serious adverse event, such as nosocomial infection. Therefore, it is important to monitor and report such events when they do occur. While similar findings have been reported with the BioStar device by NMT Medical Inc. [5, 6], these reports are the first of its kind regarding PFO or ASD closure using the Gore Cardioform devices.

## 5. Conclusions

In this retrospective analysis of recent patients who underwent percutaneous PFO or ASD closure, the incidence of postoperative fever was 13%. The symptoms typically occurred 24 to 48 hours postprocedure (average 1.25 days), and available diagnostic evaluation did not identify underlying infection. Symptoms resolved spontaneously. All cases occurred with the Gore GSO or GCA device. There were no demographic, clinical, echocardiographic, or procedural details that were associated with postprocedure fever.

There was a numerically higher incidence of AF (3 of 8, 38% vs. 10 of 54, 19%) in patients who had fever postprocedure which did not reach statistical significance (*p*=0.347). The episodes of AF occurred on average sooner (12 vs. 16 days postprocedure). All episodes of AF were self-limited and resolved. The fever response in these cases appears to be benign and self-limiting; however, further study is necessary to determine the postprocedural fever response's true incidence, etiology, and long-term prognosis.

## Figures and Tables

**Table 1:** Baseline patient characteristics.

Variables	Fever postprocedure		*p* value
	No (*n* = 54)	Yes (*n* = 8)	
Age (years)	58.0 ± 14.9	56.5 ± 9.3	0.789
Gender			0.707
Female	28 (51.9%)	3 (37.5%)	
Male	26 (48.1%)	5 (62.5%)	
Previous medical history			
HTN	24 (44.4%)	1 (12.5%)	0.086
DM	4 (7.4%)	0 (0.0%)	0.426
CAD	8 (14.8%)	1 (12.5%)	0.862
Dyslipidemia	27 (50.0%)	3 (37.5%)	0.509
OSA	17 (31.5%)	2 (25.0%)	0.711
AF	4 (7.4%)	0 (0.0%)	0.426
DVT	3 (5.6%)	0 (0.0%)	0.494
PE	5 (9.3%)	1 (12.5%)	0.772
TIA	14 (25.9%)	1 (12.5%)	0.408
Stroke	33 (61.1%)	6 (75.0%)	0.448
CKD	3 (5.6%)	0 (0.0%)	0.494
Alcohol use			
Nondrinker	14 (25.9%)	2 (25.0%)	1.000
Former	2 (3.7%)	0 (0.0%)	1.000
Moderate	37 (68.5%)	6 (75.0%)	1.000
Heavy	1 (1.9%)	0 (0.0%)	1.000
Smoking status			
Nonsmoker	33 (61.1%)	3 (37.5%)	0.262
Former	20 (37.0%)	4 (50.0%)	0.700
Current	1 (1.9%)	1 (12.5%)	0.243
Medications			
Aspirin	38 (70.4%)	5 (62.5%)	0.692
Clopidogrel	9 (16.7%)	2 (25.0%)	0.623
Coumadin	1 (1.9%)	0 (0.0%)	1.000
Rivaroxaban	1 (1.9%)	0 (0.0%)	1.000
Apixaban	9 (16.7%)	1 (12.5%)	1.000
Beta blockers	10 (18.5%)	2 (25.0%)	0.645
Calcium channel blockers	3 (5.6%)	1 (12.5%)	0.433
ACE inhibitors	10 (18.5%)	0 (0.0%)	0.333
Angiotensin II receptor blockers	11 (20.4%)	1 (12.5%)	1.000
Statins	30 (55.6%)	6 (75.0%)	0.450
Corticosteroids^∗^	10 (18.5%)	1 (12.5%)	1.000
Active chemotherapy	1 (1.9%)	0 (0.0%)	1.000
Allergies			
Nickel	1 (1.9%)	0 (0.0%)	1.000

Data are presented as mean ± SD and *n* (%). There are no significant statistical differences between patients who developed fever postprocedure and those who did not. Moderate alcohol use is described as 1 drink a day for women and up to 2 drinks a day for men. Heavy alcohol use is described as 3 on any day for women and 4 for men. HTN, hypertension; DM. diabetes mellitus, CAD, coronary artery disease; OSA, obstructive sleep apnea; AF, atrial fibrillation; DVT, deep vein thrombosis; PE, pulmonary embolism; TIA, transient ischemic attack; CKD, chronic kidney disease. ^∗^Corticosteroids include inhaled corticosteroids such as fluticasone and budesonide; topical steroids such as hydrocortisone, clobetasol; and anti-inflammatory glucocorticoid such as prednisone.

**Table 2:** Characteristics of patients with postprocedural fever.

Patient no.	1	2	3	4	5	6	7	8
*T*_max_ (°F)	NA	101	100.6	99.9	100.4	100.4	100.8	101.7
Post-op onset day	2	1	1	1	1	2	1	1
Indication	ASD	ASD	Stroke	Stroke	ASD	Stroke	Hypoxia	Stroke
Defect type	ASD	ASD	PFO	PFO	ASD	PFO	PFO	PFO
Device type	GSO	GCA	GSO	GSO	GSO	GSO	GSO	GCA
Size (mm)	30	32	25	30	30	25	30	44
AF?	N	Y	N	Y	N	N	Y	N
CBC	—	—	Low platelets	WNL	WNL	WNL	WNL	—
WBC count	—	—	9.5	8.8	9	5.8	9.2	—
Blood culture	—	—	No growth	No growth	No growth	No growth	No growth	No growth
CXR	—	—	—	Normal	Normal	Normal	Normal	—
UA	—	—	WNL	WNL	—	WNL	—	—
Leukocyte esterase	—	—	NEG	NEG	—	NEG	—	—
Nitrites	—	—	NEG	NEG	—	NEG	—	—
SARS-CoV-2	—	—	—	—	NEG	NEG	NEG	—
Influenza A/B	—	—	—	—	NEG	NEG	NEG	—
COVID-19 vaccination	N	Y	N	Y	Y	Y	Y	Y

All boxes filled with “—” indicates the labs, and/or studies have not been conducted. AF, atrial fibrillation; ASD, atrial septal defect; CBC, complete blood count; CXR, chest X-ray; GCA, Gore Cardioform ASD occluder, GSO, Gore Cardioform septal occluder; PFO, patent foramen ovale; UA; urinalysis; WBC, white blood cell count; WNL, within normal limits. NA, not available.

**Table 3:** Preprocedural echocardiographic and transcranial Doppler data.

Variables	Fever postprocedure		*p* value
	No (*n* = 54)	Yes (*n* = 8)	
Echocardiographic data			
Ejection fraction (%)	61.1 ± 5.610)	63.875 (3.643)	0.175
LA size	52.000 (14.452)	52.400 (14.571)	0.954
E/e' lateral	7.335 (2.736)	7.433 (5.074)	0.942
Biplane volume index	26.216 (6.070)	24.500 (5.753)	0.522
E/A ratio	1.090 (0.518)	1.080 (0.279)	0.966
Shunt by color	43 (82.7%)	7 (87.5%)	1.000
Atrial septal aneurysm	19 (36.5%)	6 (75.0%)	0.057
Tunnel PFO	3 (9.4%)	2 (25.0%)	0.414
Defect characterization			0.633
PFO	33 (80.5%)	6 (75.0%)	
Secundum ASD	7 (17.1%)	2 (25.0%)	
Transcranial Doppler			0.929
SLS grade 1	1 (3.2%)	0 (0.0%)	
SLS grade 2	4 (12.9%)	1 (20.0%)	
SLS grade 3	7 (22.6%)	1 (20.0%)	
SLS grade 4	16 (51.6%)	3 (60.0%)	
SLS grade 5	3 (9.7%)	0 (0.0%)	

Data are presented as mean ± SD and *n* (%). There are no significant statistical differences between patients who developed fever postprocedure and those who did not. Spencer logarithmic scale (SLS) grading was used for this transcranial Doppler study.

**Table 4:** Procedural details.

Variables	Fever postprocedure		*p* value
	No (*n* = 54)	Yes (*n* = 8)	
Indication			0.856
PFO			
Secondary prevention of stroke/TIA	30 (55.6%)	4 (50.0%)	
Hypoxia	8 (14.8%)	1 (12.5%)	
Platypnea orthodeoxia	3 (5.6%)	0 (0.0%)	
Others^∗^	4 (7.6%)	0 (0.0%)	
ASD			
Secundum ASD	7 (13.0%)	3 (37.5%)	
Iatrogenic ASD (iASD)	2 (3.7%)	0 (0.0%)	
Device type			0.500
Amplatzer septal occluder	3 (5.7%)	0 (0.0%)	
GORE cardioform ASD (GCA)	5 (9.3%)	2 (25.0%)	
GORE cardioform septal occluder (GSO)	46 (85.2%)	6 (75.0%)	
Device size (mm)			0.519
25	12 (22.2%)	2 (25.0%)	
30	35 (64.8%)	4 (50.0%)	
32	2 (3.7%)	1 (12.5%)	
35	1 (1.9%)	0 (0.0%)	
44	3 (5.6%)	1 (12.5%)	
Max ACT			0.631
Mean (SD)	289.8 (90.9)	305.6 (39.1)	
Average follow-up (months)			0.762
Mean (SD)	13.2 (9.6)	14.3 (7.6)	
Median (Q1, Q3)	11.500 (5.000, 21.250)	12.000 (10.000, 18.750)	
Range	1–37	5–26	n/a
Day of discharge			
Next day	24 (44.4%)	2 (25.0%)	0.450
Same day	28 (51.9%)	6 (75.0%)	0.276
Postprocedure ED admission	6 (11.1%)	3 (37.5%)	0.083
Type of sedation			0.328
General anesthesia	7 (12.9%)	2 (25.0%)	
Moderate sedation	47 (87.0%)	6 (75.0%)	
Year of procedure			1.000
2020	10 (18.5%)	1 (12.5%)	
2021	25 (46.3%)	4 (50.0%)	
2022	19 (35.2%)	3 (37.5%)	
Closure rate^✝^	53 (98.1%)	8 (100.0%)	1.000

Data are *n* (%). There are no significant statistical differences between patients who developed fever post-op and those who did not. ^∗^Other indications include closure for SCUBA diving, residual shunt following previous PFO closure, paradoxical embolism ^✝^Closure rate is defined by a postprocedure SLS grade 0 or 1 with Valsalva by transcranial Doppler and/or a small to no shunt size by echocardiogram. One patient had a postprocedure TCD SLS grade 3.

**Table 5:** Adverse events.

Variables	Fever postprocedure		*p* value
	No (*n* = 54)	Yes (*n* = 8)	
AF	10 (18.5%)	3 (37.5%)	0.347
Average time to develop AF (days)	16.2 (8.1)	12.0 (5.29)	0.425
GI symptoms	4 (7.4%)	1 (12.5%)	0.511
Respiratory symptoms	3 (5.6%)	2 (25.0%)	0.120
Urinary symptoms	1 (1.9%)	1 (12.5%)	0.243
Death	2 (3.7%)	0 (0.0%)	1.000

Data are presented as mean ± SD and n (%). AF, atrial fibrillation; GI, gastrointestinal.

## Data Availability

The data that support the findings of this study are not publicly available due to patient privacy concerns.
